# Utility of McGrath Video Laryngoscope in detecting unexpected Tracheal tube kinking

**DOI:** 10.12669/pjms.38.6.6582

**Published:** 2022

**Authors:** Abdullah Nisar, Faraz Shafiq

**Affiliations:** 1 Abdullah Nisar, Resident, Department of Anaesthesiology, The Aga Khan University, Stadium Road, Karachi, Pakistan; 2 Faraz Shafiq, Associate Professor, Department of Anaesthesiology, The Aga Khan University, Stadium Road, Karachi, Pakistan

**Fig.1:**
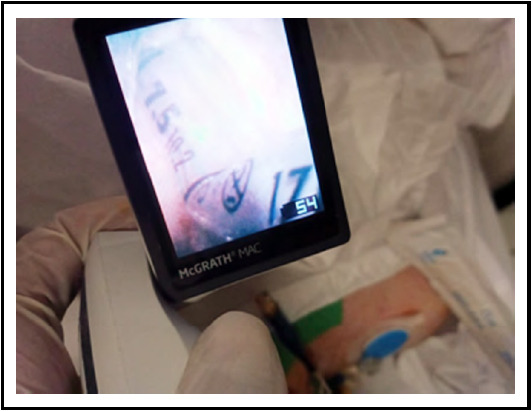
Intra-oral kinking of ETT as viewed via McGrath Video Laryngoscope.

Dear Editor,

Airway protection and maintaining patency during neurosurgical cases requiring prone positioning, is of paramount importance. Due to complexity of procedure , it becomes difficult to manipulate an airway once the surgery commences: With provision of video laryngoscopes (VDL) it becomes easier to identify airway edema and other related abnormalities.^1^ We would like to report an event of unexpected kinking of tracheal tube (TT) during resection of cerebellar mass in prone positioning, and utility of McGrath in terms of detecting that. This young female was intubated with Polyvinyl chloride cuffed TT of 7.5mm ID, fixed at 21cm. Initial ventilation after doing prone was adjusted on volume-control, where we noticed an unexpected increase in airway resistance. This was associated with delievery of sub-optimal tidal volume and up-slopping of capnograph trace. However, Oxygen saturation was remained normal. Auscultation of chest revealed bilateraly equal air entry. The situation was managed with pressure adjusted ventilation. At the end of surgery, patient was made supine, . We planned to perform the check laryngoscopy using McGrath VDL which revealed no abnormality at level of cords and correct placement of TT. However, a sharp kink was identified at 15cm mark when the blade was being retracted back. The kink was causing almost complete obstruction of the TT tube (Fig.1). Ventilation and airway pressures were drastically improved when patient was reintubated and TT was replaced.
